# Therapeutic vaccination for treatment of chronic hepatitis B

**DOI:** 10.1111/cei.13614

**Published:** 2021-06-08

**Authors:** Tamsin Cargill, Eleanor Barnes

**Affiliations:** ^1^ Peter Medawar Building for Pathogen Research Oxford University Oxford United Kingdom; ^2^ Translational Gastroenterology Unit Oxford University Oxford United Kingdom; ^3^ Oxford NIHR Biomedical Research Centre and Nuffield Department of Medicine Oxford University Oxford United Kingdom

**Keywords:** hepatitis B, immunotherapy, T cell, vaccination

## Abstract

Chronic hepatitis B infection remains a serious global health threat, contributing to a large number of deaths through liver cirrhosis and hepatocellular carcinoma. Current treatment does not eradicate disease, and therefore new treatments are urgently needed. In acute hepatitis B virus (HBV) a strong immune response is necessary to clear the virus, but in chronic infection the immune response is weakened and dysfunctional. Therapeutic vaccination describes the process of inoculating individuals with a non‐infective form of viral antigen with the aim of inducing or boosting existing HBV‐specific immune responses, resulting in sustained control of HBV infection. In this review we outline the rationale for therapeutic vaccination in chronic HBV infection, discuss previous and ongoing trials of novel HBV therapeutic vaccine candidates and outline strategies to improve vaccine efficacy going forward.

## Graphical abstract

Hepatitis B virus (HBV) is a serious global health threat. Current treatments do not cure the disease. This review summaries the potential of therapeutic vaccination as a strategy to treat chronic HBV infection.

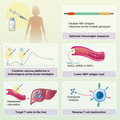

## INTRODUCTION

Chronic infection with hepatitis B virus (HBV) is a serious public health issue. With an estimated 257 million people chronically infected and an increasing incidence of hepatitis‐related deaths from cirrhosis and hepatocellular carcinoma (1), strategies to prevent, detect, treat and cure HBV are urgently needed (2).

Hepatitis B is a partially double‐stranded DNA virus that exclusively infects hepatocytes via the bile acid transporter, sodium taurocholate co‐transporting polypeptide (NTCP) (3). Infection is typically acquired through vertical transmission from mother to child at birth, via sexual transmission or exposure to blood containing infective virus (for example, re‐use of contaminated needles or unscreened blood transfusions) (1, 4, 5, 6). In the majority of individuals infected during adulthood, acute infection with HBV results in clearance of the virus by a robust immune response. Conversely, in childhood chronic infection ensues in the majority (7).

Chronic HBV has various clinical stages defined by HBV DNA titre, presence of hepatitis B e antigen (HBeAg, a secreted form of the core protein) and the presence or absence of liver inflammation measured by liver transaminase levels (8). The majority of individuals in early chronic disease are in the ‘HBeAg‐positive chronic infection’ phase, previously termed the ‘immune‐tolerant phase’, where HBV DNA is high, HBeAg is positive and liver transaminases (thought to reflect liver inflammation in HBV infection) are low. Subsequently, a period of fluctuating transaminase levels may ensue, known as ‘HBeAg‐positive chronic hepatitis’ – in some, this is followed by loss of HBeAg and seroconversion to anti‐HBeAg. ‘HBeAg‐negative chronic infection’ occurs if HBeAg lost is accompanied by decreasing HBV DNA levels and normalization of liver transaminases. Other individuals develop intermittent or persistently raised transaminases after HBeAg loss, known as ‘HBeAg‐negative chronic hepatitis’. Progression through these stages is not necessarily linear or unidirectional (8).

Nucleos(t)ide analogues, the mainstay of chronic HBV treatment, suppress viral replication and reduce the risk of liver cirrhosis and hepatocellular carcinoma (8). However, if therapy is withdrawn viral load usually rebounds, driven by unchecked transcription of HBV covalently closed circular DNA (cccDNA) that acts as a mini‐chromosome in the nucleus of infected hepatocytes. Characteristics to predict when HBV repression is likely to be maintained on withdrawal of nucleos(t)ide analogues are currently lacking. Therefore, life‐long treatment is usually required (8, 9, 10).

The ultimate goal of HBV treatment is ‘functional cure’, defined as a sustained loss of hepatitis B surface antigen (HBsAg) (11, 12). In this scenario, although HBV cccDNA remains at low levels, a functional adaptive immune response ensures suppression of viral replication without treatment, analogous to that which occurs on clearance of acute HBV (13). A strong HBV‐specific CD8 T cell response is required for HBV clearance in acute infection (14), but in chronic HBV the T cell response is dysfunctional and is not fully restored by nucleos(t)ide analogues (15). As functional cure is rarely achieved with current therapy (16), alternative treatments that can be given in shorter courses are urgently required (11, 12).

Several immunotherapies are under development to improve functional cure rates in chronic HBV, so‐called as their mechanism of action is via direct targeting of the immune response (reviewed in [17, 18, 19]). Therapeutic vaccination, the focus of this review, refers to the process of delivering HBV antigen in a non‐infective form, in order to stimulate new or augment existing HBV‐specific adaptive immune responses. This review explains our current knowledge of the T cell response towards HBV infection in order to demonstrate the immunological rational for therapeutic vaccination. It details the results of previous human trials of therapeutic vaccination for chronic HBV and outlines future directions to improve the success of vaccination strategies.

## ADAPTIVE IMMUNE RESPONSES IN ACUTE AND CHRONIC HBV INFECTION

### Adaptive immune responses are necessary for clearance of acute HBV

Studies of the immune response in acutely infected individuals that go on to resolve HBV consistently show robust CD8 and CD4 T cell responses towards regions of HBV proteins, including core, polymerase and surface (envelope) (13, 20, 21, 22, 23, 24, 25, 26, 27). *Ex‐vivo*, these cells appear functional, producing proinflammatory cytokines such as interferon (IFN)‐γ) on restimulation with recombinant HBV antigens or peptides. CD8 but not CD4 T cell depletion in HBV‐infected non‐human primates disrupts viral elimination (14), demonstrating that CD8 T cells are necessary to resolve acute infection, largely through non‐cytopathic mechanisms (28).

HBV surface‐specific B cells are also important in acute HBV infection. Anti‐hepatitis B surface antigen (HBsAg) antibodies (HBsAb) neutralize HBsAg and contribute to sustained HBsAg loss, which defines resolution of acute HBV infection (29).

### The adaptive immune response is dysfunctional in chronic HBV infection

**FIGURE 1:**
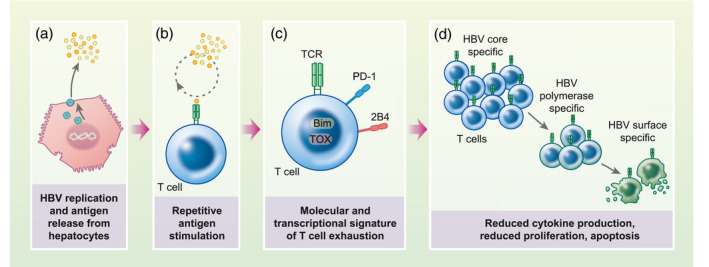
CD8 T cells are dysfunctional in chronic hepatitis B virus (HBV) infection. HBV undergoes replication in infected hepatocytes, which release HBV antigens including virions and secreted proteins. HBV‐specific T cells are repetitively stimulated by HBV antigen through their T cell receptor (TCR), which leads to the development of a molecular and transcriptional programme of T cell exhaustion, characterized by surface expression of check‐point inhibitors such as programmed cell death 1 (PD)‐1 and CD244 (2B4), mitochondrial dysfunction and transcription of *TOX* and *Bim*. As a result, HBV‐specific T cells become dysfunctional in cytokine secretion and proliferation, eventually undergoing apoptosis. HBV surface‐specific T cells are affected more severely than polymerase and core‐specific T cells. Figure created with BioRender

In contrast to people who achieve HBV clearance, chronic HBV infection is associated with dysfunctional HBV‐specific T cells (Figure 1). HBV‐specific T cells may be detected in individuals with chronic HBV infection, but are lower in frequency and less functionally responsive (30, 31, 32, 33, 34, 35, 36). Flow cytometric analysis has revealed surface up‐regulation of immune check‐point receptors such as programmed cell death 1 (PD‐1), CD244 (2B4), cytotoxic T lymphocyte antigen (CTLA)‐4 and T cell immunoglobulin and mucin domain‐containing protein 3 (Tim‐3) (30, 37, 38, 39, 40, 41). Blockade *in‐vitro* rescues the proliferative and cytokine‐secreting capacities of these cells (30, 39, 42, 43), suggesting that signalling pathways through immune check‐points might mediate T cell dysfunction. A detailed comparative analysis revealed a hierarchy of inhibitory receptor expression in chronic HBV, dominated by PD‐1 (44). Disruption of PD‐1 signalling was most successful in restoring T cell function compared to blockade of other inhibitory receptors, identifying PD‐1 as a potential target for interventions to restore HBV‐specific cellular immunity.

Driven by repetitive antigen stimulation over long periods, up‐regulation of immune check‐points in association with T cell hyporesponsiveness, known as T cell exhaustion, has also been described in other chronic viral infections and cancer (45). HBV‐specific T cells derived from the liver, the site of maximal HBV antigen expression, have higher PD‐1 expression than circulating cells (37, 44, 46), supporting the hypothesis that T cell exhaustion in HBV is driven by chronic antigen exposure. Transcriptional dysregulation of HBV‐specific T cells is similar to T cell exhaustion in other settings. For example, the transcription factor *TOX*, a central co‐ordinator of the exhausted T cell phenotype (47, 48), has recently been shown to be up‐regulated in HBV‐specific T cells during chronic infection in conjunction with PD‐1 (49). Other abnormalities include down‐regulation of the proinflammatory transcription factor *T‐bet* (50) alongside genes associated with mitochondrial metabolism (51) and up‐regulation of pro‐apoptotic transcription factors such as *Bim* (39, 52). Together, these findings support that HBV‐specific T cells in chronic infection have a transcriptional programme that co‐ordinates poor effector function, disordered metabolism and eventual apoptotic deletion.

HBV surface‐specific B cell responses are also depleted and dysfunctional in chronic HBV infection (53, 54, 55). They are inefficient at differentiating into antibody‐secreting plasma cells, which might lead to relative underproduction of HBsAbs and reduced HBsAg neutralization (53, 54). HBV surface‐specific B cells have an atypical memory phenotype and up‐regulate their surface expression of inhibitory receptors such as Fc receptor IIb and PD‐1 (53, 54). Similar to HBV‐specific T cells, their function can be partially restored by PD‐1 blockade (53, 54). Atypical memory B cells have also been described in response to repetitive antigen simulation in other chronic viral infections (56). Therefore, similar mechanisms might drive both B and T cell dysfunction in chronic HBV infection, as cells are chronically exposed to HBV antigens.

### HBV‐specific T cells have heterogeneous phenotypes in chronic infection

The extent to which HBV‐specific T cells are exhausted is, in part, related to their specificity. *Ex‐vivo* circulating HBV surface‐specific T cells are less frequent than core or polymerase targeting T cells, and are often only detectable after *in‐vitro* expansion with cognate peptides (30, 32, 33, 34, 44, 57, 58). A recent cross‐sectional study of children and adults with chronic HBV revealed that the longer an individual had been exposed to HBsAg, the lower the frequency of surface‐specific T cells (55). In addition, surface‐specific T cells have the highest PD‐1 expression (58), suggesting that they might represent a more severely exhausted phenotype prone to deletion. Differences in phenotype and function have also been observed in core and polymerase‐specific T cells. Core‐specific T cells are most numerous *ex vivo*, have a phenotype consistent with T effector memory cells and expand most efficiently *in vitro* (57, 58). The majority express high CD127 and PD‐1, consistent with a memory‐like phenotype (59). Conversely, polymerase‐specific T cells are more heterogeneous, with a proportion of cells displaying a naive phenotype (57, 58). They expand less efficiently *in vitro* and express higher KLRG1 and *EOMES*, consistent with a more severely exhausted phenotype (57). Together, these data suggest that functional recovery of surface and polymerase‐restricted T cells with immunotherapy may be more challenging than for core‐specific cells.

### HBV‐specific T cells in the liver are important for HBV control

The local immune response in the liver, the site of HBV infection, may significantly impact upon T cell function (34, 60). The majority of HBV‐specific T cells in the liver have a resident memory phenotype, implying that they do not recirculate. They express high levels of PD‐1 and negatively correlate with HBV DNA titre, suggesting that they are involved in viral control. On restimulation, these cells produce proinflammatory cytokines including IFN‐γ and tumour necrosis factor (TNF)‐α, as well as interleukin (IL)‐2 to HBV core, polymerase and surface peptides, in contrast to the poorly responsive HBV‐specific T cells in the periphery. However, intrahepatic T cells in chronic HBV show reduced expression of Ki‐67 and granzyme B, suggesting that their proliferative and cytotoxic functions might be impaired (46). Despite these cells having high PD‐1 expression they appear to retain some functionally, suggesting that in the liver, at least, PD‐1 might not be representative of a fully exhausted T cell. During acute HBV, PD‐1 is also increased on HBV‐specific T cells and has a positive correlation with resolution of infection (61). Together, this suggests that intrahepatic T cells in the liver are functional despite high PD‐1 expression and their augmentation might be a target to induce sustained immune control of chronic HBV. The differences in peripheral and intrahepatic HBV‐specific T cells are clearly relevant for the reporting of T cell phenotype and function in clinical studies, and for trials of immunotherapies efficacy end‐points that do not rely primarily upon the evaluation of T cell function in blood are required.

### HBV‐specific T cell dysfunction is not irreversible and might be restored by therapeutic vaccination

Once chronic HBV infection is established most people will remain infected for life. However, after sustained HBV DNA suppression in people treated with peginterferon or nucleos(t)ide analogues, a small proportion achieve functional cure with sustained HBsAg loss (16). In the majority, nucleos(t)ide analogue therapy only transiently improves T cell function (15, 62, 63, 64). However, in individuals achieving HBsAg loss, HBV‐specific T cell function is improved despite retaining high expression of exhaustion‐related molecules such as TOX and PD‐1 (49, 65). In people with resolved infection, circulating T cell responses are comparable to those who have cleared acute HBV infection (65) and are retained in the liver long term (46). These data show that despite T cells retaining a molecular signature of exhaustion, chronic HBV‐associated T cell dysfunction is not irreversible. Therapeutic vaccination aims to induce new or augment existing HBV‐specific T cell responses by directly providing non‐infective HBV antigen in chronically infected people.

## PREVIOUS CLINICAL TRIALS OF THERAPEUTIC VACCINATION IN CHRONIC HBV

During the last 20 years multiple studies have assessed therapeutic vaccine candidates for chronic HBV therapy (Supporting information, Tables S1–S6). Although none have been successful in inducing functional cure so far, some regimes have shown promise in improving HBV viral parameters and HBV‐specific immune responses.

### Recombinant vaccines

Initial investigations focused upon recombinant protein vaccines comprising the small and medium HBV surface proteins (Pre‐S2 and S, Supporting information, Table S1). In healthy uninfected individuals, recombinant HBsAg vaccines induced HBsAbs and CD4^+^ T cell responses (66, 67, 68) and were established as prophylactic vaccines to prevent HBV infection.

However, when investigated in individuals with untreated chronic HBV infection, although vaccination induced detectable surface‐specific T cell responses in a subset of individuals (69) there was no significant difference between vaccinated and unvaccinated groups in achieving HBV DNA suppression, HBeAg loss or HBsAg loss (70, 71, 72, 73, 74, 75). In an attempt to improve immunogenicity, vaccines combining HBsAg with hepatitis B immunoglobulin were tested in untreated individuals, but ultimately this had similarly disappointing results (Supporting information, Table S2, (76, 77, 78, 79, 80)). Even in combination with nucleos(t)ide analogues or interferon treatment, vaccine therapy showed no additional benefit over nucleos(t)ide analogues alone (81, 82, 83, 84, 85, 86, 87). Another recombinant vaccine CVI‐HBV‐002 delivering the long, medium and small HBV surface proteins (surface/pre‐S2/Pre‐s1 regions) with adjuvant (L‐pampo) is currently in Phase II clinical trials in chronic HBV patients, although results are yet to be reported (88).

One probable reason for the failure of these trials is that vaccine strategies for the effective prevention of HBV infection are likely to differ significantly from strategies required for HBV cure once chronic HBV infection is established. Although in healthy people HBsAg induces HBsAbs which block viral entry and prevent infection, the effects on T cell induction and immune restoration in the chronic setting using protein vaccines alone are likely to be minimal.

Another possible reason as to why these early trials of recombinant HBsAg vaccine were not efficacious is that a large proportion of participants had HBeAg‐positive disease, associated with high HBV antigen levels. High antigen loads have been proposed as a barrier to the successful rescue of tolerized T cells by therapeutic vaccination (89). Subsequently, two small studies have assessed recombinant HBsAg vaccine efficacy in individuals with e antigen‐negative disease, suppressed HBV DNA and low antigen loads (HBsAg 100–1000 IU/ml). The first retrospectively assessed the effect of vaccination on preventing relapse of viral replication after withdrawal from nucleos(t)ide therapy, but found no benefit of vaccination (90). More promising results were obtained by administration of recombinant vaccine in people with low HBsAg levels, which was associated with further reductions in HBsAg titres and undetectable HBsAg in some participants. However, there was no prospective control arm in this study (91). Larger randomized studies are warranted to assess whether recombinant vaccines have a role in treating individuals with low baseline HBsAg levels.

Finally, the ineffectiveness of recombinant HBsAg vaccines may have been because they only delivered a single HBV antigen, whereas in acute infection immune responses specific to multiple HBV antigens are present (14). To overcome this issue, a vaccination strategy delivering HBV core antigen (HBcAg) in addition to HBsAg has been developed. A randomized trial of recombinant HBsAg and HBcAg in individuals with chronic HBV showed a significant reduction in HBV DNA when compared to peginterferon treatment alone, 24 weeks after therapy completion (92). Although these results are promising, HBsAg loss was not reported.

### Lipopeptide epitope‐base vaccine

In order to develop vaccines with superior T cell immunogenicity, an epitope‐based lipopeptide vaccine CY‐1889 was developed (Supporting information, Table S3). This delivered a single HBV CD8 T cell core epitope (Core_18–27_), known to be present in people who controlled acute HBV (20). Although this vaccine induced HBV core‐specific CD8 T cells in healthy volunteers (93), when administered to chronically infected individuals CD8 T cell responses were of lower magnitude and no reductions in HBV DNA were observed (94). A major limitation of an immune strategy based on single epitopes is that this will be limited to individuals who carry the HLA alleles that restrict that particular epitope and will generate a very narrow immune response that may select for immune escape variants.

### DNA vaccines

Alternative vaccine approaches designed to optimize CD8 T cell responses also include DNA vaccines alone or in combination with MVA vectored vaccines in heterologous prime‐boost strategies (Supporting information, Table S4). Unfortunately, when compared to no treatment or nucleos(t)ide analogue therapy, these regimes did not improve virological parameters (95, 96, 97, 98, 99, 100). Broadening the HBV antigens included within the immunogen to core and polymerase, in addition to surface, also failed to improve efficacy (101, 102). Enzyme‐linked immunospot (ELISPOT) assays showed detectable but low T cell responses in people with chronic HBV in response to vaccination, suggesting that these regimes were unable to overcome the HBV‐specific T cell hyporesponsiveness characteristic of chronic infection.

### Yeast‐derived vaccines

More recent therapeutic vaccine candidates have been designed to induce multi‐specific HBV responses by incorporating several HBV antigens or epitopes. GS‐4774, a yeast‐derived vaccine, includes HBsAg, HBcAg and hepatitis B X, chosen for their relative conservation across HBV genotypes (103). Although HBV‐specific T cell responses were induced in healthy volunteers after vaccination, in the majority of individuals the response magnitude was low–moderate (below 200 spot‐forming units per million cells) (103). Subsequent randomized trials of individuals with chronic HBV have had disappointing results (Supporting information, Table S5). In a trial conducted in 178 individuals with chronic infection with suppressed HBV DNA and relatively low HBsAg levels (mean 2.9 log_10_ IU/ml), nucleos(t)ide analogue therapy alone was compared to the addition of GS‐4774 vaccination. Although HBsAg titres decreased in all groups, larger declines of 0.5 log_10_ IU/ml or more were detected in only three of the 178 participants, all of whom were in groups receiving the highest dose of vaccine (104). End‐of‐study immunological assessment showed detectable but low‐magnitude T cell responses to vaccination (104). Subsequently, GS‐4774, in combination with nucleos(t)ide analogue tenofovir, was compared to tenofovir alone in 195 participants with chronic HBV infection, this time with raised HBV DNA levels and higher HBsAg tires (mean 3.7 log_10_ IU/ml) (105). No participant lost HBsAg after 48 weeks and decreases in HBsAg titre were not significantly different between groups (105). One reason that this vaccine might not be efficacious in these trials is that it is not able to induce T cell responses of adequate magnitude for immune protection in established chronic HBV infection. Even in healthy subjects, only low–moderate T cell responses were induced in the majority (103) and this might not be enough to overcome T cell tolerance associated with chronic HBV.

### Adenoviral vectored vaccines

Another promising vaccine candidate, TG‐1050, comprises a human adenoviral vector encoding core, polymerase and surface HBV antigens within its immunogen. Its safety and immunogenicity were studied in a recent Phase I clinical trial either as a single dose or in homologous prime‐boost vaccination strategies (Supporting information, Table S6 [106]). Participants had undetectable HBV DNA on enrolment on nucleos(t)ide therapy with raised HBsAg titres, ranging from 2.2 to 4.6 log_10_ IU/ml. A year after the first vaccination, HBsAg loss of more than 0.2 log_10_ IU/ml was achieved in 19% of vaccinated (*n* = 7) and 8% of unvaccinated (*n* = 1) participants. T cell responses to vaccination, measured after *in‐vitro* expansion, showed increased responses in some, but not all, participants tested (106). In keeping with data that core‐specific responses retain the most functionality in chronic HBV (57, 58), core‐specific T cell responses were most recoverable in response to vaccination, followed by polymerase and surface‐specific responses (106). Unfortunately, as this vaccine has not been tested in healthy individuals, it is not clear whether the poor T cell responses after vaccination seen in this trial are due to poor immunogenicity of the vaccine itself or as a consequence of chronic HBV infection. Another potential disadvantage of using a human adenoviral vector is that anti‐adenovirus antibodies, if present at baseline, may impair immunogenicity, as has been the case in previous studies (107, 108).

### Summary

Thus far, attempts at therapeutic vaccination for HBV have been ineffective in reliably inducing functional cure in people with chronic HBV. The persistence of HBV‐specific T cell hyporesponsiveness and high baseline HBsAg load of participants, together with limited T cell immunogenicity of the vaccine candidates themselves, are possible reasons that may have hampered the success of these vaccine candidates. The following section explores possible strategies to overcome these barriers in future vaccine development.

## FUTURE DIRECTIONS TO IMPROVE HBV THERAPEUTIC VACCINATION STRATEGIES

**FIGURE 2:**
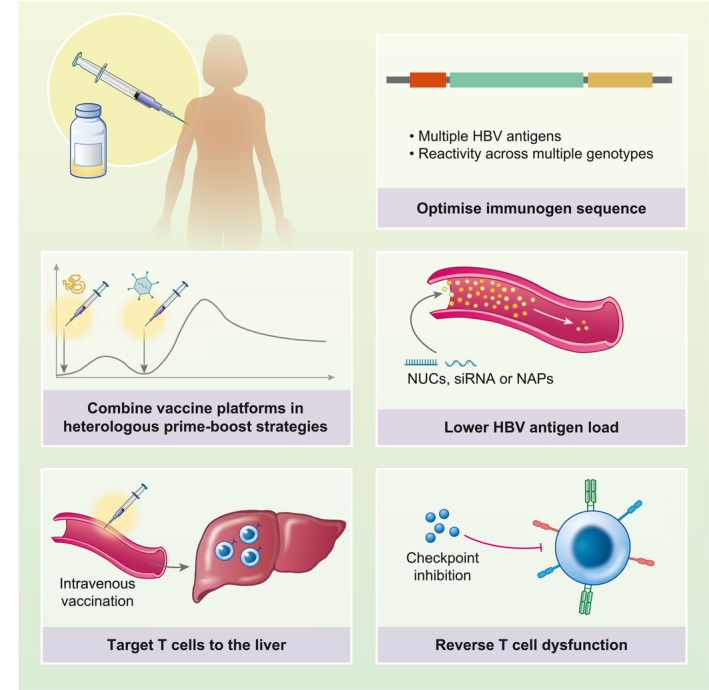
Strategies to improve therapeutic vaccination for chronic hepatitis B virus (HBV) infection. Strategies include (i) vaccinating individuals with lower HBV antigen loads or reducing antigenic load prior to vaccination with nucleos(t)ide therapy (NUCs), small inhibitory RNA (siRNA) or nucleic acid polymers (NAPs); (ii) reversing T cell dysfunction with concomitant treatment with check‐point inhibitors; (iii) optimization of vaccine immunogen by including multiple HBV antigens; (iv) combining vaccine platforms in heterologous prime‐boost strategies; and (v) targeting T cells to the liver via, for example, intravenous vaccination. Figure created with BioRender

Several strategies have promise to improve the efficacy of therapeutic vaccination in chronic HBV (Figure 2). Consistently, HBV vaccine candidates do not seem to induce strong HBV‐specific T cell responses when administered to people with chronic HBV, even when they are shown to be immunogenic in healthy individuals (66, 67, 68, 93, 103, 109, 110, 111, 112). Potential approaches to improve therapeutic vaccination, therefore, all attempt to augment vaccine‐induced T cell responses and overcome the HBV‐specific T cell dysfunction associated with chronic infection.

### HBV optimizing patient selection – lowering HBV antigen load

High levels of HBsAg are thought to contribute to the HBV‐specific T cell dysfunction in chronic HBV (55). Quantitative HBsAg measures serum HBsAg transcribed from HBV cccDNA, as well as HBV DNA integrated into the host genome (113). In patients with suppressed circulating HBV DNA on nucleos(t)ide therapy quantitative HBsAg levels remain elevated, despite reduced HBV replication. In these individuals, integrated HBV DNA is the most important source of HBsAg (114).

Continually raised HBsAg levels is one mechanism contributing to the sustained suppression of HBV surface‐specific immune responses, which hampers therapeutic vaccine efficacy. One strategy is to enrol groups of patients with lower baseline HBV antigen load, whose HBV‐specific T cells are more susceptible to functional recovery. This is supported by animal models of chronic HBV in which therapeutic vaccination is more successful when HBsAg titres are low (115, 116, 117) and in people with chronic HBV stopping nucleos(t)ide therapy, in whom low HBsAg titres at time of nucleos(t)ide withdrawal are associated with subsequent HBsAg loss during follow‐up (118). Pretreatment with therapies to reduce HBsAg, such as small interference RNA (siRNA) or nucleic acid polymers (NAPs) (119, 120, 121), might be useful to reduce HBsAg levels prior to therapeutic vaccination. These therapies would need to supress HBsAg production from both cccDNA and integrated HBV DNA. The reduction of HBsAg prior to vaccination might also enable younger people with HBV infection to be treated, in whom the frequency of surface‐specific T cells appears to be higher and T cell function might be more recoverable (55).

### Concomitant reversal of T cell dysfunction with check‐point inhibition

Another strategy to boost the efficacy of therapeutic vaccination is by concurrent treatment with check‐point inhibitors that block the PD‐1 signalling pathway. The effectiveness of this approach was first demonstrated in the woodchuck model of chronic hepatitis B, in which check‐point inhibitor in combination with therapeutic vaccine was able to induce surface antigen loss (122). In a small open‐label Phase I trial in adults with HBeAg‐negative chronic infection on nucleos(t)ide therapy, low‐dose check‐point inhibitor therapy alone or in combination with GS‐4774 vaccine was found to be safe, and induced HBsAg loss in one participant (123). A Phase II clinical trial has recently opened to recruitment to assess therapeutic HBV vaccines ChAdOx1‐HBV and MVA‐HBV in combination with check‐point inhibitor (124). The results from this trial will be important to determine whether this approach is effective in humans.

### Optimization of vaccine immunogens

Strong T cell responses to multiple HBV epitopes are important (14) in the clearance of acute HBV. Most of our knowledge of HBV epitopes is skewed towards HLA‐A*02‐restricted epitopes, although it is clear that HBV‐infected people with different human leucocyte antigen (HLA) alleles recognize other HBV epitopes (35, 125). Peptide vaccine FP‐02.2 (HepTcell) incorporates nine HBV T cell epitopes in conserved regions of the HBV genome, which might increase its chances of being immunogenic among populations with various HLA types. Early results of a Phase I study (NCT02496897) confirmed safety when administered to participants with chronic HBV, and a Phase 2 trial is now recruiting (NCT04684914) (126, 127, 128). An advantage of vaccines that target conserved areas of the HBV genome is that they are more likely to be able to induce immunogenicity among different genotypes. Further work to discover immunoprotective HBV epitopes among different HBV genotypes in people with diverse HLA haplotypes is another approach to inform better vaccine design.

### Selection of vaccine vector

To give the very best chance of inducing the strong T cell responses required to clear HBV infection, vaccine candidates should use platforms known to induce T cell responses of the highest magnitude. Several vaccination approaches designed to induce high‐magnitude HBV T cell responses are currently being evaluated in clinical trials, including vaccine VBI‐2601, which utilizes a virus‐like particle platform (ACTRN12619001210167) or administration of DNA vaccine JNJ‐64300535 using electroporation technology (NCT03463369) (129, 130).

Other strategies known to induce strong CD8 T cell responses use vectors given in heterologous prime‐boost strategies and have been successful in inducing functional cure in animal models of chronic hepatitis B (115, 131, 132). Vaccination with particulate HBsAg and HBcAg has been successful in inducing HBsAg loss in a murine model of chronic HBV infection when combined with a booster vaccination using a modified vaccinia virus Ankara (MVA)‐encoding HBV core and surface regions (115). This approach of combining HBsAg and HBcAg with MVA boost vaccination requires further investigation in human studies.

Simian adenoviral vector prime MVA vectors boost vaccine strategies are among the most potent vaccine combinations for inducing T cell responses in humans (133, 134). There are several advantages to these platforms that might be advantageous for HBV therapy. First, they are able to encode large immunogens, making it possible for almost the entire HBV genome to be incorporated into the vaccine. This increases the probability that T cell responses towards multiple epitopes across individuals with a variety of HLA types will be induced. In addition, they are able to induce both T and B cell responses in the same vector and their manufacture to good manufacturing practice standards is well established.

Therapeutic vaccines using adenoviral and MVA vectors have been shown to induce high‐magnitude polyfunctional HBV‐specific T cell responses in preclinical studies (135). Two clinical trials are currently ongoing to assess the safety and immunogenicity of chimpanzee adenoviral and MVA vectored HBV vaccine candidates in healthy volunteers and individuals with chronic HBV (GSK3528869A, NCT03866187 and ChAdOx1‐HBV, NCT04297917 [136, 137]).

Another potential vaccine platform that might be efficacious for HBV therapy are mRNA vaccines, which induce robust antibody and T cell responses and have had recent success in prophylactic vaccination in SARS‐CoV‐2 (reviewed in [138]). Although no HBV therapeutic vaccines have employed the mRNA platform to date, it is an attractive option for future development.

### Targeting T cells to the liver

Maximizing effective HBV‐specific T cells at the site of infection might also be critical in inducing HBV functional cure. Resident memory T cells have been shown to be key for local control of HBV in the liver (46). A prime–pull vaccination strategy which employs intramuscular delivery of a simian adenoviral vector to prime T cells in the periphery, followed by an intravenous boost of MVA‐vectored vaccine to pull T cells into the liver, was shown to be effective in protecting mice from the liver stage of malaria (139). This strategy also increased the magnitude of HBV‐specific T cells in the liver at peak time‐point after boost MVA vaccination in mice (140), but its effectiveness at inducing HBV functional cure remains to be tested. These data support that, for hepatotrophic pathogens, it is critical to understand the T cell response in the liver in order to optimize vaccine efficacy. This has not been attempted routinely in human vaccine studies due to the invasiveness of liver biopsy. Recently, however, fine‐needle aspiration (FNA) has been shown to be a safe alternative to biopsy that enables serial assessment of local liver immunology (60). Future clinical studies should consider FNA as routine to evaluate the intrahepatic response to vaccination which can inform and optimize vaccine development.

## CONCLUSIONS

T cells are key to effective resolution of acute HBV infection. In chronic infection the T cell response is dysfunctional; however, a subset of individuals can go on to develop long‐term immunological control of the virus, signalled by loss of HBsAg. Therapeutic vaccination aims to restore the HBV‐specific immune response and has demonstrated efficacy in inducing functional cure in animal models of chronic HBV. Despite disappointing results in human clinical trials thus far, therapeutic vaccination remains a promising immunotherapeutic strategy. Optimizing T cell responses by selecting maximally immunogenic vaccine vectors, vaccination routes and immunoprotective epitopes, together with agents that reduce HBV T cell dysfunction such as immune check‐point inhibitors, have real potential to overcome the barriers to immunological control imposed by chronic infection.

## CONFLICT OF INTERESTS

The authors have no financial conflicts of interest. Ellie Barnes is a named inventor and Ellie Barnes and Tamsin Cargill are contributors on a patent application describing ChAdOx1‐HBV vaccine reported in this manuscript (International Application no. PCT/GB2018/050948).

## AUTHOR CONTRIBUTIONS

Tamsin Cargill and Ellie Barnes jointly wrote and reviewed the article prior to submission.

## Supporting information

Table S1‐S6

## Data Availability

Data sharing is not applicable to this article as no new data were created or analyzed in this study.

## References

[1] World Health Organization . Global hepatitis report, 2017. https://www.who.int/hepatitis/publications/global‐hepatitis‐report2017/en/. Accessed 3 Oct 2019.

[2] World Health Organization (WHO) . Global health sector strategy on viral hepatitis 2016–2021. 2016. https://www.who.int/hepatitis/strategy2016‐2021/ghss‐hep/en/. Accessed 3 Oct 2019.

[3] Yan H , Zhong G , Xu G , He W , Jing Z , Gao Z , et al. Sodium taurocholate cotransporting polypeptide is a functional receptor for human hepatitis B and D virus. eLife. 2012;13:e00049. 10.7554/eLife.00049 PMC3485615 23150796

[4] Beasley RP , Trepo C , Stevens CE , Szmuness W . The e antigen and vertical transmission of hepatitis B surface antigen. Am J Epidemiol. 1977;105:94–8. 835566 10.1093/oxfordjournals.aje.a112370

[5] Chaudhuri AK , Follett EA , Burrell CJ . Anti‐e and vertical transmission of hepatitis B surface antigen. BMJ. 1977;2:1416–7. 10.1136/bmj.2.6099.1416-d PMC1632357 589241

[6] Okada K , Kamiyama I , Inomata M , Imai M , Miyakawa Y , Mayumi M . e antigen and anti‐e in the serum of asymptomatic carrier mothers as indicators of positive and negative transmission of hepatitis B virus to their infants. N Engl J Med. 1976;294:746–9. 943694 10.1056/NEJM197604012941402

[7] Edmunds WJ , Medley GF , Nokes DJ , Hall AJ , Whittle HC . The influence of age on the development of the hepatitis B carrier state. Proc Biol Sci. 1993;253:197–201. 8397416 10.1098/rspb.1993.0102

[8] European Association for the Study of the Liver (EASL) . Clinical Practice Guidelines on the management of hepatitis B virus infection. J Hepatol. 2017;67:370–98. 28427875 10.1016/j.jhep.2017.03.021

[9] Papatheodoridis G , Vlachogiannakos I , Cholongitas E , Wursthorn K , Thomadakis C , Touloumi G , et al. Discontinuation of oral antivirals in chronic hepatitis B: a systematic review. Hepatology. 2016;63:1481–92. 27100145 10.1002/hep.28438

[10] Berg T , Simon K‐G , Mauss S , Schott E , Heyne R , Klass DM , et al. Long‐term response after stopping tenofovir disoproxil fumarate in non‐cirrhotic HBeAg‐negative patients – FINITE study. J Hepatol. 2017;67:918–24. 28736139 10.1016/j.jhep.2017.07.012

[11] Lok AS , Zoulim F , Dusheiko G , Ghany MG . Hepatitis B cure: from discovery to regulatory approval. J Hepatol. 2017;67:847–61. 28778687 10.1016/j.jhep.2017.05.008

[12] Ning Q , Wu DI , Wang G‐Q , Ren H , Gao Z‐L , Hu P , et al. Roadmap to functional cure of chronic hepatitis B: an expert consensus. J Viral Hepat. 2019;26:1146–55. 31087479 10.1111/jvh.13126

[13] Rehermann B , Ferrari C , Pasquinelli C , Chisari FV . The hepatitis B virus persists for decades after patients’ recovery from acute viral hepatitis despite active maintenance of a cytotoxic T‐lymphocyte response. Nat Med. 1996;2:1104–8. 8837608 10.1038/nm1096-1104

[14] Thimme R , Wieland S , Steiger C , Ghrayeb J , Reimann KA , Purcell RH , et al. CD8(+) T cells mediate viral clearance and disease pathogenesis during acute hepatitis B virus infection. J Virol. 2003;77:68–76. 12477811 10.1128/JVI.77.1.68-76.2003 PMC140637

[15] Boni C , Penna A , Bertoletti A , Lamonaca V , Rapti I , Missale G , et al. Transient restoration of anti‐viral T cell responses induced by lamivudine therapy in chronic hepatitis B. J Hepatol. 2003;39:595–605. 12971971 10.1016/s0168-8278(03)00292-7

[16] Lok AS , Zoulim F , Dusheiko G , Chan HLY , Buti M , Ghany MG , et al. Durability of hepatitis B surface antigen loss with nucleotide analogue and peginterferon therapy in patients with chronic hepatitis B. Hepatol Commun. 2020;4:8–20. 31909352 10.1002/hep4.1436 PMC6939500

[17] Gehring AJ , Protzer U . Targeting innate and adaptive immune responses to cure chronic HBV infection. Gastroenterology. 2019;156:325–37. 30367834 10.1053/j.gastro.2018.10.032

[18] Alexopoulou A , Vasilieva L , Karayiannis P . New approaches to the treatment of chronic hepatitis B. J Clin Med. 2020;9:3187. 10.3390/jcm9103187 PMC7601587 33019573

[19] Maini MK , Burton AR . Restoring, releasing or replacing adaptive immunity in chronic hepatitis B. Nat Rev Gastroenterol Hepatol. 2019;16:662–75. 31548710 10.1038/s41575-019-0196-9

[20] Maini MK , Boni C , Ogg GS , King AS , Reignat S , Lee CK , et al. Direct *ex vivo* analysis of hepatitis B virus‐specific CD8(+) T cells associated with the control of infection. Gastroenterology. 1999;117:1386–96. 10579980 10.1016/s0016-5085(99)70289-1

[21] Ferrari C , Penna A , Bertoletti A , Valli A , Antoni AD , Giuberti T , et al. Cellular immune response to hepatitis B virus‐encoded antigens in acute and chronic hepatitis B virus infection. J Immunol. 1990;145:3442–9. 2230128

[22] Ferrari C , Bertoletti A , Penna A , Cavalli A , Valli A , Missale G , et al. Identification of immunodominant T cell epitopes of the hepatitis B virus nucleocapsid antigen. J Clin Invest. 1991;88:214–22. 1711541 10.1172/JCI115280 PMC296022

[23] Penna A , Chisari FV , Bertoletti A , Missale G , Fowler P , Giuberti T , et al. Cytotoxic T lymphocytes recognize an HLA‐A2‐restricted epitope within the hepatitis B virus nucleocapsid antigen. J Exp Med. 1991;174:1565–70. 1720813 10.1084/jem.174.6.1565 PMC2119048

[24] Bertoletti A , Ferrari C , Fiaccadori F , Penna A , Margolskee R , Schlicht HJ , et al. HLA class I‐restricted human cytotoxic T cells recognize endogenously synthesized hepatitis B virus nucleocapsid antigen. Proc Natl Acad Sci USA. 1991;88:10445–9. 1660137 10.1073/pnas.88.23.10445 PMC52945

[25] Bertoletti A , Chisari FV , Penna A , Guilhot S , Galati L , Missale G , et al. Definition of a minimal optimal cytotoxic T‐cell epitope within the hepatitis B virus nucleocapsid protein. J Virol. 1993;67:2376–80. 7680391 10.1128/jvi.67.4.2376-2380.1993 PMC240403

[26] Missale G , Redeker A , Person J , Fowler P , Guilhot S , Schlicht HJ , et al. HLA‐A31‐ and HLA‐Aw68‐restricted cytotoxic T cell responses to a single hepatitis B virus nucleocapsid epitope during acute viral hepatitis. J Exp Med. 1993;177:751–62. 7679709 10.1084/jem.177.3.751 PMC2190933

[27] Nayersina R , Fowler P , Guilhot S , Missale G , Cerny A , Schlicht HJ , et al. HLA A2 restricted cytotoxic T lymphocyte responses to multiple hepatitis B surface antigen epitopes during hepatitis B virus infection. J Immunol. 1993;150:4659–71. 7683326

[28] Guidotti LG , Rochford R , Chung J , Shapiro M , Purcell R , Chisari FV . Viral clearance without destruction of infected cells during acute HBV infection. Science. 1999;284:825–9. 10221919 10.1126/science.284.5415.825

[29] Song JE , Kim DY . Diagnosis of hepatitis B. Ann Transl Med. 2016;4:338. 27761442 10.21037/atm.2016.09.11 PMC5066055

[30] Boni C , Fisicaro P , Valdatta C , Amadei B , Di Vincenzo P , Giuberti T , et al. Characterization of hepatitis B virus (HBV)‐specific T‐cell dysfunction in chronic HBV infection. J Virol. 2007;81:4215–25. 17287266 10.1128/JVI.02844-06 PMC1866111

[31] Das A , Hoare M , Davies N , Lopes AR , Dunn C , Kennedy PTF , et al. Functional skewing of the global CD8 T cell population in chronic hepatitis B virus infection. J Exp Med. 2008;205:2111–24. 18695005 10.1084/jem.20072076 PMC2526205

[32] Maini MK , Boni C , Lee CK , Larrubia JR , Reignat S , Ogg GS , et al. The role of virus‐specific CD8(+) cells in liver damage and viral control during persistent hepatitis B virus infection. J Exp Med. 2000;191:1269–80. 10770795 10.1084/jem.191.8.1269 PMC2193131

[33] Reignat S , Webster GJM , Brown D , Ogg GS , King A , Seneviratne SL , et al. Escaping high viral load exhaustion: CD8 cells with altered tetramer binding in chronic hepatitis B virus infection. J Exp Med. 2002;195:1089–101. 11994415 10.1084/jem.20011723 PMC2193712

[34] Chang JJ , Thompson AJV , Visvanathan K , Kent SJ , Cameron PU , Wightman F , et al. The phenotype of hepatitis B virus‐specific T cells differ in the liver and blood in chronic hepatitis B virus infection. Hepatology. 2007;46:1332–40. 17924445 10.1002/hep.21844

[35] Tan AT , Loggi E , Boni C , Chia A , Gehring AJ , Sastry KSR , et al. Host ethnicity and virus genotype shape the hepatitis B virus‐specific T‐cell repertoire. J Virol. 2008;82:10986–97. 18799575 10.1128/JVI.01124-08 PMC2573267

[36] Webster GJM , Reignat S , Brown D , Ogg GS , Jones L , Seneviratne SL , et al. Longitudinal analysis of CD8+ T cells specific for structural and nonstructural hepatitis B virus proteins in patients with chronic hepatitis B: implications for immunotherapy. J Virol. 2004;78:5707–19. 15140968 10.1128/JVI.78.11.5707-5719.2004 PMC415806

[37] Fisicaro P , Valdatta C , Massari M , Loggi E , Biasini E , Sacchelli L , et al. Antiviral intrahepatic T‐cell responses can be restored by blocking programmed death‐1 pathway in chronic hepatitis B. Gastroenterology. 2010;138:682–93, 693.e1‐4. 19800335 10.1053/j.gastro.2009.09.052

[38] Raziorrouh B , Schraut W , Gerlach T , Nowack D , Grüner NH , Ulsenheimer A , et al. The immunoregulatory role of CD244 in chronic hepatitis B infection and its inhibitory potential on virus‐specific CD8+ T‐cell function. Hepatology. 2010;52:1934–47. 21064032 10.1002/hep.23936

[39] Schurich A , Khanna P , Lopes AR , Han KJ , Peppa D , Micco L , et al. Role of the coinhibitory receptor cytotoxic T lymphocyte antigen‐4 on apoptosis‐prone CD8 T cells in persistent hepatitis B virus infection. Hepatology. 2011;53:1494–503. 21360567 10.1002/hep.24249

[40] Nebbia G , Peppa D , Schurich A , Khanna P , Singh HD , Cheng Y , et al. Upregulation of the Tim‐3/galectin‐9 pathway of T cell exhaustion in chronic hepatitis B virus infection. PLOS ONE. 2012;7:e47648. 23112829 10.1371/journal.pone.0047648 PMC3480425

[41] Park J‐J , Wong DK , Wahed AS , Lee WM , Feld JJ , Terrault N , et al. Hepatitis B virus‐specific and global T‐cell dysfunction in chronic hepatitis B. Gastroenterology. 2016;150:684–695.e5. 26684441 10.1053/j.gastro.2015.11.050 PMC4766024

[42] Wu W , Shi Y , Li S , Zhang Y , Liu Y , Wu Y , et al. Blockade of Tim‐3 signaling restores the virus‐specific CD8^+^ T‐cell response in patients with chronic hepatitis B. Eur J Immunol. 2012;42:1180–91. 22539292 10.1002/eji.201141852

[43] Fisicaro P , Valdatta C , Massari M , Loggi E , Ravanetti L , Urbani S , et al. Combined blockade of programmed death‐1 and activation of CD137 increase responses of human liver T cells against HBV, but not HCV. Gastroenterology. 2012;143:1576–1585.e4. 22929808 10.1053/j.gastro.2012.08.041

[44] Bengsch B , Martin B , Thimme R . Restoration of HBV‐specific CD8+ T cell function by PD‐1 blockade in inactive carrier patients is linked to T cell differentiation. J Hepatol. 2014;61:1212–9. 25016223 10.1016/j.jhep.2014.07.005

[45] Blank CU , Haining WN , Held W , Hogan PG , Kallies A , Lugli E , et al. Defining ‘T cell exhaustion’. Nat Rev Immunol. 2019;19:665–74. 31570879 10.1038/s41577-019-0221-9 PMC7286441

[46] Pallett LJ , Davies J , Colbeck EJ , Robertson F , Hansi N , Easom NJW , et al. IL‐2high tissue‐resident T cells in the human liver: Sentinels for hepatotropic infection. J Exp Med. 2017;214:1567–80. 28526759 10.1084/jem.20162115 PMC5461007

[47] Khan O , Giles JR , McDonald S , Manne S , Ngiow SF , Patel KP , et al. TOX transcriptionally and epigenetically programs CD8+ T cell exhaustion. Nature. 2019;571:211–8. 31207603 10.1038/s41586-019-1325-x PMC6713202

[48] Alfei F , Kanev K , Hofmann M , Wu M , Ghoneim HE , Roelli P , et al. TOX reinforces the phenotype and longevity of exhausted T cells in chronic viral infection. Nature. 2019;571:265–9. 31207605 10.1038/s41586-019-1326-9

[49] Heim K , Binder B , Sagar , Wieland D , Hensel N , Llewellyn‐Lacey S , et al. TOX defines the degree of CD8+ T cell dysfunction in distinct phases of chronic HBV infection. Gut. 2020. doi:10.1136/gutjnl-2020-322404. PMC8292571 33097558

[50] Kurktschiev PD , Raziorrouh B , Schraut W , Backmund M , Wächtler M , Wendtner C‐M , et al. Dysfunctional CD8+ T cells in hepatitis B and C are characterized by a lack of antigen‐specific T‐bet induction. J Exp Med. 2014;211:2047–59. 25225458 10.1084/jem.20131333 PMC4172217

[51] Fisicaro P , Barili V , Montanini B , Acerbi G , Ferracin M , Guerrieri F , et al. Targeting mitochondrial dysfunction can restore antiviral activity of exhausted HBV‐specific CD8 T cells in chronic hepatitis B. Nat Med. 2017;23:327–36. 28165481 10.1038/nm.4275

[52] Lopes AR , Kellam P , Das A , Dunn C , Kwan A , Turner J , et al. Bim‐mediated deletion of antigen‐specific CD8 T cells in patients unable to control HBV infection. J Clin Invest. 2008;118:1835–45. 18398508 10.1172/JCI33402 PMC2289792

[53] Burton AR , Pallett LJ , McCoy LE , Suveizdyte K , Amin OE , Swadling L , et al. Circulating and intrahepatic antiviral B cells are defective in hepatitis B. J Clin Invest. 2018;128:4588–603. 30091725 10.1172/JCI121960 PMC6159997

[54] Salimzadeh L , Le Bert N , Dutertre C‐A , Gill US , Newell EW , Frey C , et al. PD‐1 blockade partially recovers dysfunctional virus‐specific B cells in chronic hepatitis B infection. J Clin Invest. 2018;128:4573–87. 30084841 10.1172/JCI121957 PMC6159957

[55] Le Bert N , Gill US , Hong M , Kunasegaran K , Tan DZM , Ahmad R , et al. Effects of hepatitis B Surface antigen on virus‐specific and global T Cells in patients with chronic hepatitis B virus infection. Gastroenterology. 2020;159:652–64. 32302614 10.1053/j.gastro.2020.04.019

[56] Portugal S , Obeng‐Adjei N , Moir S , Crompton PD , Pierce SK . Atypical memory B cells in human chronic infectious diseases: an interim report. Cell Immunol. 2017;321:18–25. 28735813 10.1016/j.cellimm.2017.07.003 PMC5732066

[57] Schuch A , Salimi Alizei E , Heim K , Wieland D , Kiraithe MM , Kemming J , et al. Phenotypic and functional differences of HBV core‐specific versus HBV polymerase‐specific CD8+ T cells in chronically HBV‐infected patients with low viral load. Gut. 2019;68:905–15. 30622109 10.1136/gutjnl-2018-316641

[58] Hoogeveen RC , Robidoux MP , Schwarz T , Heydmann L , Cheney JA , Kvistad D , et al. Phenotype and function of HBV‐specific T cells is determined by the targeted epitope in addition to the stage of infection. Gut. 2019;68:893–904. 30580250 10.1136/gutjnl-2018-316644

[59] Wieland D , Kemming J , Schuch A , Emmerich F , Knolle P , Neumann‐Haefelin C , et al. TCF1+ hepatitis C virus‐specific CD8+ T cells are maintained after cessation of chronic antigen stimulation. Nat Commun. 2017;3:15050. 10.1038/ncomms15050 PMC5418623 28466857

[60] Gill US , Pallett LJ , Thomas N , Burton AR , Patel AA , Yona S , et al. Fine needle aspirates comprehensively sample intrahepatic immunity. Gut. 2019;68:1493–503. 30487267 10.1136/gutjnl-2018-317071 PMC6691856

[61] Zhang Z , Zhang J‐Y , Wherry EJ , Jin B , Xu B , Zou Z‐S , et al. Dynamic programmed death 1 expression by virus‐specific CD8 T cells correlates with the outcome of acute hepatitis B. Gastroenterology. 2008;134:1938–49, 1949.e1‐3. 18455515 10.1053/j.gastro.2008.03.037

[62] Boni C , Bertoletti A , Penna A , Cavalli A , Pilli M , Urbani S , et al. Lamivudine treatment can restore T cell responsiveness in chronic hepatitis B. J Clin Invest. 1998;102:968–75. 9727065 10.1172/JCI3731 PMC508962

[63] Boni C , Penna A , Ogg GS , Bertoletti A , Pilli M , Cavallo C , et al. Lamivudine treatment can overcome cytotoxic T‐cell hyporesponsiveness in chronic hepatitis B: new perspectives for immune therapy. Hepatology. 2001;33:963–71. 11283861 10.1053/jhep.2001.23045

[64] Malacarne F , Webster G , Reignat S , Gotto J , Behboudi S , Burroughs A , et al. Tracking the source of the hepatitis B virus‐specific CD8 T cells during lamivudine treatment. J Infect Dis. 2003;187:679–82. 12599086 10.1086/368369

[65] Boni C , Laccabue D , Lampertico P , Giuberti T , Viganò M , Schivazappa S , et al. Restored function of HBV‐specific T cells after long‐term effective therapy with nucleos(t)ide analogues. Gastroenterology. 2012;143:963–973.e9. 22796241 10.1053/j.gastro.2012.07.014

[66] Desombere I , Gijbels Y , Verwulgen A , Leroux‐Roels G . Characterization of the T cell recognition of hepatitis B surface antigen (HBsAg) by good and poor responders to hepatitis B vaccines. Clin Exp Immunol. 2000;122:390–9. 11122245 10.1046/j.1365-2249.2000.01383.x PMC1905794

[67] Honorati MC , Dolzani P , Mariani E , Piacentini A , Lisignoli G , Ferrari C , et al. Epitope specificity of Th0/Th2 CD4+ T‐lymphocyte clones induced by vaccination with rHBsAg vaccine. Gastroenterology. 1997;112:2017–27. 9178695 10.1053/gast.1997.v112.pm9178695

[68] Min WP , Kamikawaji N , Mineta M , Tana T , Kashiwagi S , Sasazuki T . Identification of an epitope for T‐cells correlated with antibody response to hepatitis B surface antigen in vaccinated humans. Hum Immunol. 1996;46:93–9. 8727207 10.1016/0198-8859(96)00009-2

[69] Couillin I , Pol S , Mancini M , Driss F , Bréchot C , Tiollais P , et al. Specific vaccine therapy in chronic hepatitis B: induction of T cell proliferative responses specific for envelope antigens. J Infect Dis. 1999;180:15–26. 10353856 10.1086/314828

[70] Pol S , Driss F , Michel ML , Nalpas B , Berthelot P , Brechot C . Specific vaccine therapy in chronic hepatitis B infection. Lancet. 1994;344:342. 10.1016/s0140-6736(94)91384-6 7914291

[71] Pol S , Nalpas B , Driss F , Michel M‐L , Tiollais P , Denis J , et al. Efficacy and limitations of a specific immunotherapy in chronic hepatitis B. J Hepatol. 2001;34:917–21. 11451177 10.1016/s0168-8278(01)00028-9

[72] Pata C , Yazar A , Konca K , Bilgiç G , Eskandari G , Oztürk C . The effect of recombinant hepatitis B vaccine therapy in chronic hepatitis B infection. Turk J Gastroenterol. 2002;13:6–10. 16378267

[73] Yalcin K , Acar M , Degertekin H . Specific hepatitis B vaccine therapy in inactive HBsAg carriers: a randomized controlled trial. Infection. 2003;31:221–5. 14562945 10.1007/s15010-003-3187-1

[74] Dikici B , Kalayci AG , Ozgenc F , Bosnak M , Davutoglu M , Ece A , et al. Therapeutic vaccination in the immunotolerant phase of children with chronic hepatitis B infection. Pediatr Infect Dis J. 2003;22:345–9. 12690275 10.1097/01.inf.0000059443.49414.8b

[75] Dikici B , Bosnak M , Ucmak H , Dagli A , Ece A , Haspolat K . Failure of therapeutic vaccination using hepatitis B surface antigen vaccine in the immunotolerant phase of children with chronic hepatitis B infection. J Gastroenterol Hepatol. 2003;18:218–22. 12542609 10.1046/j.1440-1746.2003.02950.x

[76] Wen YM , Wu XH , Hu DC , Zhang QP , Guo SQ . Hepatitis B vaccine and anti‐HBs complex as approach for vaccine therapy. Lancet. 1995;345:1575–6. 10.1016/s0140-6736(95)91126-x 7791465

[77] Yao X , Zheng B , Zhou J , Xu D‐Z , Zhao K , Sun S‐H , et al. Therapeutic effect of hepatitis B surface antigen‐antibody complex is associated with cytolytic and non‐cytolytic immune responses in hepatitis B patients. Vaccine. 2007;25:1771–9. 17224217 10.1016/j.vaccine.2006.11.019

[78] Xu D‐Z , Zhao K , Guo L‐M , Chen X‐Y , Wang H‐F , Zhang J‐M , et al. A randomized controlled phase IIb trial of antigen–antibody immunogenic complex therapeutic vaccine in chronic hepatitis B patients. PLOS ONE. 2008;3:e2565. 18596958 10.1371/journal.pone.0002565 PMC2430617

[79] Wang X‐Y , Zhang X‐X , Yao X , Jiang J‐H , Xie Y‐H , Yuan Z‐H , et al. Serum HBeAg sero‐conversion correlated with decrease of HBsAg and HBV DNA in chronic hepatitis B patients treated with a therapeutic vaccine. Vaccine. 2010;28:8169–74. 20937312 10.1016/j.vaccine.2010.09.093

[80] Xu D‐Z , Wang X‐Y , Shen X‐L , Gong G‐Z , Ren H , Guo L‐M , et al. Results of a phase III clinical trial with an HBsAg‐HBIG immunogenic complex therapeutic vaccine for chronic hepatitis B patients: experiences and findings. J Hepatol. 2013;59:450–6. 23669281 10.1016/j.jhep.2013.05.003

[81] Dahmen A , Herzog‐Hauff S , Böcher WO , Galle PR , Löhr HF . Clinical and immunological efficacy of intradermal vaccine plus lamivudine with or without interleukin‐2 in patients with chronic hepatitis B. J Med Virol. 2002;66:452–60. 11857521 10.1002/jmv.2165

[82] Demirtürk N , Usluer G , Ozgünes I , Colak H , Kartal ED , Dinçer S . Comparison of different treatment combinations for chronic hepatitis B infection. J Chemother. 2002;14:285–9. 12120884 10.1179/joc.2002.14.3.285

[83] Helvaci M , Kizilgunesler A , Kasirga E , Ozbal E , Kuzu M , Sozen G . Efficacy of hepatitis B vaccination and interferon‐alpha‐2b combination therapy versus interferon‐alpha‐2b monotherapy in children with chronic hepatitis B. J Gastroenterol Hepatol. 2004;19:785–91. 15209626 10.1111/j.1440-1746.2004.03358.x

[84] Horiike N , Akbar SMF , Michitaka K , Joukou K , Yamamoto K , Kojima N , et al. *In vivo* immunization by vaccine therapy following virus suppression by lamivudine: a novel approach for treating patients with chronic hepatitis B. J Clin Virol. 2005;32:156–61. 15653419 10.1016/j.jcv.2004.07.004

[85] Vandepapelière P , Lau GKK , Leroux‐Roels G , Horsmans Y , Gane E , Tawandee T , et al. Therapeutic vaccination of chronic hepatitis B patients with virus suppression by antiviral therapy: a randomized, controlled study of co‐administration of HBsAg/AS02 candidate vaccine and lamivudine. Vaccine. 2007;25:8585–97. 18031872 10.1016/j.vaccine.2007.09.072

[86] Ishikawa T , Kakumu S . Combination therapy with lamivudine and HB vaccine on chronic hepatitis B. Hepatol Res. 2007;37:S62–66. 17627638 10.1111/j.1872-034X.2007.00107.x

[87] Hoa PTL , Huy NT , Thu LT , Nga CN , Nakao K , Eguchi K , et al. Randomized controlled study investigating viral suppression and serological response following pre‐S1/pre‐S2/S vaccine therapy combined with lamivudine treatment in HBeAg‐positive patients with chronic hepatitis B. Antimicrob Agents Chemother. 2009;53:5134–40. 19770281 10.1128/AAC.00276-09 PMC2786371

[88] CHA Vaccine Institute Co., Ltd . A Randomized, Double‐blinded, Placebo‐controlled, Parallel, Multicenter, Phase 2b Study to Evaluate the Efficacy and Safety of CVI‐HBV‐002 in Patients With Chronic Hepatitis B Taking Tenofovir Disoproxil Fumarate/Tenofovir Disoproxil. NCT04289987. ClinialTrails.gov; 2020. https://clinicaltrials.gov/ct2/show/. Accessed 16 Mar 2021.

[89] Dembek C , Protzer U , Roggendorf M . Overcoming immune tolerance in chronic hepatitis B by therapeutic vaccination. Curr Opin Virol. 2018;30:58–67. 29751272 10.1016/j.coviro.2018.04.003

[90] Lee YB , Lee J‐H , Kim YJ , Yoon J‐H , Lee H‐S . The effect of therapeutic vaccination for the treatment of chronic hepatitis B virus infection. J Med Virol. 2015;87:575–82. 25611586 10.1002/jmv.24091

[91] Lai M‐W , Hsu C‐W , Lin C‐L , Chien R‐N , Lin W‐R , Chang C‐S , et al. Multiple doses of hepatitis B recombinant vaccine for chronic hepatitis B patients with low surface antigen levels: a pilot study. Hepatol Int. 2018;12:456–64. 30088198 10.1007/s12072-018-9890-x

[92] Al Mahtab M , Akbar SMF , Aguilar JC , Guillen G , Penton E , Tuero A , et al. Treatment of chronic hepatitis B naive patients with a therapeutic vaccine containing HBs and HBc antigens (a randomized, open and treatment controlled phase III clinical trial). PLOS ONE. 2018;13:e0201236. 30133478 10.1371/journal.pone.0201236 PMC6104936

[93] Vitiello A , Ishioka G , Grey HM , Rose R , Farness P , LaFond R , et al. Development of a lipopeptide‐based therapeutic vaccine to treat chronic HBV infection. I. Induction of a primary cytotoxic T lymphocyte response in humans. J Clin Invest. 1995;95:341–9. 7814635 10.1172/JCI117662 PMC295437

[94] Heathcote J , McHutchison J , Lee S , Tong M , Benner K , Minuk G , et al. A pilot study of the CY‐1899 T‐cell vaccine in subjects chronically infected with hepatitis B virus. The CY1899 T Cell Vaccine Study Group. Hepatology. 1999;30:531–6. 10421664 10.1002/hep.510300208

[95] Mancini‐Bourgine M , Fontaine H , Scott‐Algara D , Pol S , Bréchot C , Michel M‐L . Induction or expansion of T‐cell responses by a hepatitis B DNA vaccine administered to chronic HBV carriers. Hepatology. 2004;40:874–82. 15382173 10.1002/hep.20408

[96] Mancini‐Bourgine M , Fontaine H , Bréchot C , Pol S , Michel M‐L . Immunogenicity of a hepatitis B DNA vaccine administered to chronic HBV carriers. Vaccine. 2006;24:4482–9. 16310901 10.1016/j.vaccine.2005.08.013

[97] Cavenaugh JS , Awi D , Mendy M , Hill AVS , Whittle H , McConkey SJ . Partially randomized, non‐blinded trial of DNA and MVA therapeutic vaccines based on hepatitis B virus surface protein for chronic HBV infection. PLOS ONE. 2011;6:e14626. 21347224 10.1371/journal.pone.0014626 PMC3039644

[98] Yang F‐Q , Yu Y‐Y , Wang G‐Q , Chen J , Li J‐H , Li Y‐Q , et al. A pilot randomized controlled trial of dual‐plasmid HBV DNA vaccine mediated by in vivo electroporation in chronic hepatitis B patients under lamivudine chemotherapy. J Viral Hepat. 2012;19:581–93. 22762143 10.1111/j.1365-2893.2012.01589.x

[99] Fontaine H , Kahi S , Chazallon C , Bourgine M , Varaut A , Buffet C , et al. Anti‐HBV DNA vaccination does not prevent relapse after discontinuation of analogues in the treatment of chronic hepatitis B: a randomised trial – ANRS HB02 VAC‐ADN. Gut. 2015;64:139–47. 24555998 10.1136/gutjnl-2013-305707

[100] Yang F‐Q , Rao G‐R , Wang G‐Q , Li Y‐Q , Xie Y , Zhang Z‐Q , et al. Phase IIb trial of *in vivo* electroporation mediated dual‐plasmid hepatitis B virus DNA vaccine in chronic hepatitis B patients under lamivudine therapy. World J Gastroenterol. 2017;23:306–17. 28127204 10.3748/wjg.v23.i2.306 PMC5236510

[101] Yoon SK , Seo YB , Im SJ , Bae SH , Song MJ , You CR , et al. Safety and immunogenicity of therapeutic DNA vaccine with antiviral drug in chronic HBV patients and its immunogenicity in mice. Liver Int. 2015;35:805–15. 24620920 10.1111/liv.12530

[102] Yang S‐H , Lee C‐G , Park S‐H , Im S‐J , Kim Y‐M , Son J‐M , et al. Correlation of antiviral T‐cell responses with suppression of viral rebound in chronic hepatitis B carriers: a proof‐of‐concept study. Gene Ther. 2006;13:1110–7. 16525482 10.1038/sj.gt.3302751

[103] Gaggar A , Coeshott C , Apelian D , Rodell T , Armstrong BR , Shen G , et al. Safety, tolerability and immunogenicity of GS‐4774, a hepatitis B virus‐specific therapeutic vaccine, in healthy subjects: a randomized study. Vaccine. 2014;32:4925–31. 25045824 10.1016/j.vaccine.2014.07.027

[104] Lok AS , Pan CQ , Han S‐H , Trinh HN , Fessel WJ , Rodell T , et al. Randomized phase II study of GS‐4774 as a therapeutic vaccine in virally suppressed patients with chronic hepatitis B. J Hepatol. 2016;65:509–16. 27210427 10.1016/j.jhep.2016.05.016

[105] Boni C , Janssen HLA , Rossi M , Yoon SK , Vecchi A , Barili V , et al. Combined GS‐4774 and tenofovir therapy can improve HBV‐specific T‐cell responses in patients with chronic hepatitis. Gastroenterology. 2019;157:227–241.e7. 30930022 10.1053/j.gastro.2019.03.044

[106] Zoulim F , Fournier C , Habersetzer F , Sprinzl M , Pol S , Coffin CS , et al. Safety and immunogenicity of the therapeutic vaccine TG1050 in chronic hepatitis B patients: a phase 1b placebo‐controlled trial. Hum Vaccin Immunother. 2020;16:388–99. 31373537 10.1080/21645515.2019.1651141 PMC7158919

[107] Churchyard GJ , Morgan C , Adams E , Hural J , Graham BS , Moodie Z , et al. A phase IIA randomized clinical trial of a multiclade HIV‐1 DNA prime followed by a multiclade rAd5 HIV‐1 vaccine boost in healthy adults (HVTN204). PLOS ONE. 2011;6:e21225. 21857901 10.1371/journal.pone.0021225 PMC3152265

[108] Frahm N , DeCamp AC , Friedrich DP , Carter DK , Defawe OD , Kublin JG , et al. Human adenovirus‐specific T cells modulate HIV‐specific T cell responses to an Ad5‐vectored HIV‐1 vaccine. J Clin Invest. 2012;122:359–67. 22201684 10.1172/JCI60202 PMC3248307

[109] Xu D‐Z , Huang K‐L , Zhao K , Xu L‐F , Shi N , Yuan Z‐H , et al. Vaccination with recombinant HBsAg‐HBIG complex in healthy adults. Vaccine. 2005;23:2658–64. 15780449 10.1016/j.vaccine.2004.10.040

[110] Betancourt AA , Delgado CAG , Estévez ZC , Martínez JC , Ríos GV , Aureoles‐Roselló SRM , et al. Phase I clinical trial in healthy adults of a nasal vaccine candidate containing recombinant hepatitis B surface and core antigens. Int J Infect Dis. 2007;11:394–401. 17257877 10.1016/j.ijid.2006.09.010

[111] Tacket CO , Roy MJ , Widera G , Swain WF , Broome S , Edelman R . Phase 1 safety and immune response studies of a DNA vaccine encoding hepatitis B surface antigen delivered by a gene delivery device. Vaccine. 1999;17:2826–9. 10438052 10.1016/s0264-410x(99)00094-8

[112] Roy MJ , Wu MS , Barr LJ , Fuller JT , Tussey LG , Speller S , et al. Induction of antigen‐specific CD8+ T cells, T helper cells, and protective levels of antibody in humans by particle‐mediated administration of a hepatitis B virus DNA vaccine. Vaccine 2000;19:764–78. 11115698 10.1016/s0264-410x(00)00302-9

[113] Cornberg M , Wong VW‐S , Locarnini S , Brunetto M , Janssen HLA , Chan HL‐Y . The role of quantitative hepatitis B surface antigen revisited. J Hepatol. 2017;66:398–411. 27575311 10.1016/j.jhep.2016.08.009

[114] Tu T , Budzinska MA , Shackel NA , Urban S . HBV DNA Integration: molecular mechanisms and clinical implications. Viruses. 2017;9:75. 10.3390/v9040075 PMC5408681 28394272

[115] Backes S , Jäger C , Dembek CJ , Kosinska AD , Bauer T , Stephan A‐S , et al. Protein‐prime/modified vaccinia virus Ankara vector‐boost vaccination overcomes tolerance in high‐antigenemic HBV‐transgenic mice. Vaccine. 2016;34:923–32. 26776470 10.1016/j.vaccine.2015.12.060

[116] Chiale C , Yarovinsky TO , Mason SW , Madina BR , Menon M , Krady MM , et al. Modified alphavirus‐vesiculovirus hybrid vaccine vectors for homologous prime‐boost immunotherapy of chronic hepatitis B. Vaccines. 2020;8:279. 10.3390/vaccines8020279 PMC7349932 32517032

[117] Michler T , Kosinska AD , Festag J , Bunse T , Su J , Ringelhan M , et al. Knockdown of virus antigen expression increases therapeutic vaccine efficacy in high‐titer hepatitis B virus carrier mice. Gastroenterology. 2020;158:1762–1775.e9. 32001321 10.1053/j.gastro.2020.01.032

[118] Jeng W‐J , Chen Y‐C , Chien R‐N , Sheen I‐S , Liaw Y‐F . Incidence and predictors of hepatitis B surface antigen seroclearance after cessation of nucleos(t)ide analogue therapy in hepatitis B e antigen‐negative chronic hepatitis B. Hepatology. 2018;68:425–34. 29108132 10.1002/hep.29640

[119] Al‐Mahtab M , Bazinet M , Vaillant A . Safety and efficacy of nucleic acid polymers in monotherapy and combined with immunotherapy in treatment‐naive Bangladeshi patients with HBeAg+ chronic hepatitis B infection. PLOS ONE. 2016;11:e0156667. 27257978 10.1371/journal.pone.0156667 PMC4892580

[120] Bazinet M , Pântea V , Cebotarescu V , Cojuhari L , Jimbei P , Albrecht J , et al. Safety and efficacy of REP 2139 and pegylated interferon alfa‐2a for treatment‐naive patients with chronic hepatitis B virus and hepatitis D virus co‐infection (REP 301 and REP 301‐LTF): a non‐randomised, open‐label, phase 2 trial. Lancet Gastroenterol Hepatol. 2017;2:877–89. 28964701 10.1016/S2468-1253(17)30288-1

[121] Wooddell CI , Yuen M‐F , Chan HL‐Y , Gish RG , Locarnini SA , Chavez D , et al. RNAi‐based treatment of chronically infected patients and chimpanzees reveals that integrated hepatitis B virus DNA is a source of HBsAg. Sci Transl Med. 2017;9:eaan0241. 28954926 10.1126/scitranslmed.aan0241 PMC5830187

[122] Liu J , Zhang E , Ma Z , Wu W , Kosinska A , Zhang X , et al. Enhancing virus‐specific immunity *in vivo* by combining therapeutic vaccination and PD‐L1 blockade in chronic hepadnaviral infection. PLOS Pathog. 2014;10:e1003856. 24391505 10.1371/journal.ppat.1003856 PMC3879364

[123] Gane E , Verdon DJ , Brooks AE , Gaggar A , Nguyen AH , Subramanian GM , et al. Anti‐PD‐1 blockade with nivolumab with and without therapeutic vaccination for virally suppressed chronic hepatitis B: a pilot study. J Hepatol. 2019;71:900–7. 31306680 10.1016/j.jhep.2019.06.028

[124] Vaccitech Limited . A Phase 1b/2a, open‐label study to evaluate the safety, tolerability and immunogenicity of VTP‐300 with or without nivolumab in participants with chronic hepatitis B infection. NCT04778904. ClinialTrails.gov; 2021. https://clinicaltrials.gov/ct2/show/NCT04778904. Accessed 16 Mar 2021

[125] Lumley S , Noble H , Hadley MJ , Callow L , Malik A , Chua YY , et al. Hepitopes: a live interactive database of HLA class I epitopes in hepatitis B virus. Wellcome Open Res. 2016;15:9. 10.12688/wellcomeopenres.9952.1 PMC5142601 27976751

[126] Lim Y‐S , Mutimer D , Heo J , Tak WY , Rosenberg W , Jang BK , et al. Abstract PS‐078. A phase 1b evaluation of HepTcell HBV‐specific immunotherapy in nuc‐controlled, eAg negative chronic HBV infection. J Hepatol. 2019;70:e50.

[127] Altimmune . NCT02496897 Phase I safety and immunogenicity of FP‐02.2 in chronic hepatitis B. 2019. https://clinicaltrials.gov/ct2/show/NCT02496897. Accessed 2 Jan 2021.

[128] Altimmune, Inc . HepT cell immunotherapy in patients with inactive chronic hepatitis B (CHB) NCT04684914. 2020. https://clinicaltrials.gov/ct2/show/NCT04684914. Accessed 11 Feb 2021.

[129] Brii Biosciences . A study to evaluate safety, tolerability, and antiviral activity of BRII‐179 (VBI‐2601) among subjects with chronic hepatitis B. 2019. https://anzctr.org.au/Trial/Registration/TrialReview.aspx?ACTRN=12619001210167. Accessed 12 Jan 2021.

[130] Janssen Sciences Ireland UC . A first‐in‐human study to evaluate safety, tolerability, reactogenicity, and immunogenicity of JNJ‐64300535, a DNA vaccine, administered by electroporation‐mediated intramuscular injection, in participants with chronic hepatitis B who are on stable nucleos(t)ide therapy and virologically suppressed. 2018. https://clinicaltrials.gov/ct2/show/NCT03463369. Accessed 12 Jan 2021.

[131] Kosinska AD , Johrden L , Zhang E , Fiedler M , Mayer A , Wildner O , et al. DNA prime‐adenovirus boost immunization induces a vigorous and multifunctional T‐cell response against hepadnaviral proteins in the mouse and woodchuck model. J Virol. 2012;86:9297–310. 22718818 10.1128/JVI.00506-12 PMC3416123

[132] Kosinska AD , Zhang E , Johrden L , Liu J , Seiz PL , Zhang X , et al. Combination of DNA prime – adenovirus boost immunization with entecavir elicits sustained control of chronic hepatitis B in the woodchuck model. PLOS Pathog. 2013;9:e1003391. 23785279 10.1371/journal.ppat.1003391 PMC3681757

[133] Vitelli A , Folgori A , Scarselli E , Colloca S , Capone S , Nicosia A . Chimpanzee adenoviral vectors as vaccines – challenges to move the technology into the fast lane. Expert Rev Vaccines. 2017;16:1241–52. 29047309 10.1080/14760584.2017.1394842

[134] Capone S , Brown A , Hartnell F , Sorbo MD , Traboni C , Vassilev V , et al. Optimising T cell (re)boosting strategies for adenoviral and modified vaccinia Ankara vaccine regimens in humans. NPJ Vaccines. 2020;5:94. 33083029 10.1038/s41541-020-00240-0 PMC7550607

[135] Chinnakannan SK , Cargill TN , Donnison TA , Ansari MA , Sebastian S , Lee LN , et al. The design and development of a multi‐HBV antigen encoded in chimpanzee adenoviral and modified vaccinia Ankara viral vectors; a novel therapeutic vaccine strategy against HBV. Vaccines. 2020;8:184. 10.3390/vaccines8020184 PMC7348829 32295168

[136] Vaccitech Limited . NCT04297917 A Phase 1 monotherapy study to evaluate the safety, tolerability and immunogenicity of vaccination with candidate chimpanzee adenovirus‐vectored HepB virus vaccine ChAdOx1 HBV in healthy participants and participants with chronic HepB infection. 2020. https://clinicaltrials.gov/ct2/show/NCT04297917. Accessed 2 Jan 2021.

[137] GlaxoSmithKline . Safety, efficacy, immunogenicity study of GSK Biologicals’ HBV viral vector and adjuvanted proteins vaccine (GSK3528869A) in adult patients with chronic hepatitis B infection. 2019. https://clinicaltrials.gov/ct2/show/NCT03866187. Accessed 12 Jan 2021.

[138] Cagigi A , Loré K . Immune responses induced by mRNA vaccination in mice, monkeys and humans. Vaccines. 2021;9:61. 33477534 10.3390/vaccines9010061 PMC7831080

[139] Gola A , Silman D , Walters AA , Sridhar S , Uderhardt S , Salman AM , et al. Prime and target immunization protects against liver‐stage malaria in mice. Sci Transl Med. 2018;10:eaap9128. doi:10.1126/scitranslmed.aap9128. 30257955

[140] Narayan S , Cargill T , Chinnakannan S , Lee LL , Hutchings C , Klenerman P , et al. SAT464. Optimising delivery of therapeutic hepatitis B vaccines to induce resident memory T cells in the liver. J Hepatol. 2020;73:S886.

