# Supplementary material for: Therapeutic vaccination for treatment of chronic hepatitis B
Source: Clin Exp Immunol. 2021 Jun 8;205(2):106–18. doi: 10.1111/cei.13614 (PMC8274149; doi:10.1111/cei.13614)
Supplement: Supplementary file 1 — Table S1‐S6 [file CEI-205-106-s001.docx]

**Table S1 Recombinant vaccines**

| **Study** | **Study type** | **N** | **Intervention** | **Control group** | **HBV antigens within vaccine** | **Age group** | **Population** | **FU** | **Virologic assessment** | **Immunologic assessment** |
| --- | --- | --- | --- | --- | --- | --- | --- | --- | --- | --- |
| (Pol et al., 1994) | Non-randomised | 32 | Gen Hevac B | No control group | Pre-S2, S | Adult | Chronic hepatitis, unknown HBeAg status | 26 | **HBV DNA suppression**: occurred in n=10/32 (31%) of vaccinated individuals  **HBeAg loss:** not reported **HBeAg seroconversion:** not reported **HBsAg loss:** not reported | Not reported |
| (Pol et al., 2001) | Randomised | 170 | Gen Hevac B/ Recombivax | No treatment | Pre-S2, S | Adult | HBeAg positive or negative chronic infection or chronic hepatitis | 52 | **HBV DNA suppression:** no significant difference between groups **HBeAg loss:** no significant difference between groups **HBeAg seroconversion:** no significant difference between groups **HBsAg loss:** not reported | **HBs antibody levels:** increased in n= 7/27 (26%) after vaccination, undetectable if unvaccinated. **Proliferation assay**: Positive CD4 T cell responses in n=5/27 (19%) after vaccination, undetectable if unvaccinated. |
| (Pata et al., 2002) | Non-randomised | 29 | Gen Hevac B/ Recombivax | No treatment | Pre-S2, S | Adult | HBeAg positive or negative chronic infection or chronic hepatitis | 52 | **HBV DNA suppression:** significant reduction between baseline and end of study in vaccinated group but not unvaccinated group **HBeAg loss:** not reported **HBeAg seroconversion:** n=4/11 (36%) in vaccinated group **HBsAg loss:** not reported | Not reported |
| (Yalcin et al., 2003) | Randomised | 47 | Gen Hevac B | No treatment | Pre-S2, S | Adult | HBeAg positive chronic infection | 52 | **HBV DNA suppression:** no significant difference between groups **HBeAg loss:** no significant difference between groups  **HBeAg seroconversion:** no significant difference between groups **HBsAg loss:** none | Not reported |
| (Dikici et al., 2003b) | Randomised | 74 | Gen Hevac B | No treatment | Pre-S2, S | Child | HBeAg positive chronic infection | 52 | **HBV DNA suppression:** no significant difference between groups **HBeAg loss:** n= 0/43 (0%) in vaccinated group, n=1/31 (3%) in control group  **HBeAg seroconversion:** Nil **HBsAg loss:** not reported | Not reported |
| (Dikici et al., 2003a) | Randomised | 51 | Gen Hevac B | No treatment | Pre-S2, S | Child | HBeAg positive chronic infection | 52 | **HBV DNA suppression:** no significant difference between groups **HBeAg loss:** no significant difference between groups  **HBeAg seroconversion:** nil **HBsAg loss: n=**1/23 (4%) in vaccinated group, n= 1/28 (4%) in control group | Not reported |

| **Study** | **Study type** | **N** | **Intervention** | **Control group** | **HBV antigens within vaccine** | **Age group** | **Population** | **FU** | **Virologic assessment** | **Immunologic assessment** |
| --- | --- | --- | --- | --- | --- | --- | --- | --- | --- | --- |
| (Dahmen et al., 2002) | Non-randomised | 25 | ENGERIX-B with NUCs | NUC | Pre-S2, S | Adult | HBeAg positive or negative chronic hepatitis | 26 | **HBV DNA suppression:** n=4/29 (14%) in vaccinated group  **HBeAg loss:** not reported **HBeAg seroconversion:** not reported **HBsAg loss:** not reported | **ELISpot assay (ex-vivo):** baseline responses to HBV core and surface increased in vaccination group after therapy.  **Proliferation assay:** Positive CD4 T cell responses in n=10/14 (71%) at baseline and n=8/12 (67%) after therapy in vaccination group and n=4/11 (36%) in unvaccinated group. |
| (Demirtürk et al., 2002) | Non-randomised | 29 | Gen Hevac B with IFN | NUC + IFN | Pre-S2, S | Adult | HBeAg positive or negative chronic infection or chronic hepatitis | 52 | **HBV DNA suppression:** together with ALT normalisation n=3/10 (30%) in vaccinated group and n=11/14 (79%) in combined control groups  **HBeAg loss:** n=3/3 (100%) in vaccinated group and n=1/9 (11%) in combined control groups **HBeAg seroconversion:** not reported **HBsAg loss:** nil | Not reported |
| (Helvaci et al., 2004) | Randomised | 50 | Gen Hevac B with IFN | IFN | Pre-S2, S | Child | HBeAg positive chronic infection or chronic hepatitis | 60 | **HBV DNA suppression:**  no significant difference between groups at end of study **HBeAg loss:** not reported **HBeAg seroconversion:** no significant difference between groups **HBsAg loss:** n=1/25 (4%) in vaccinated and control group | Not reported |
| (Horiike et al., 2005) | Non-randomised | 72 | HBsAg with NUC | NUC | S | Adult | HBeAg positive or negative chronic hepatitis | 52 | **HBV DNA suppression:** Within HBeAg positive subgroup, significantly higher HBV DNA suppression in vaccinated group (n=9/9, 100%) compared to control group (n=15/31, 48%) **HBeAg loss:** not reported  **HBeAg seroconversion:** Within HBeAg positive subgroup, significantly higher HBeAg seroconversion in vaccinated group (n=5/9, 56%) compared to control group (n=5/31, 15%) **HBsAg loss:** not reported | Not reported |
| (Vandepapelière et al., 2007) | Randomised | 195 | HBsAg with NUC | NUC | S | Adult | HBeAg positive chronic hepatitis | 52 | **HBV DNA suppression:** no significant difference between groups **HBeAg loss:** no significant difference between groups **HBeAg seroconversion:** no significant difference between groups **HBsAg loss:** not reported | **HBs antibody levels:** detectable in 83% of vaccination group  **Proliferation assay and ICS:** No detectable responses at baseline in either group. In vaccinated group, cellular responses peaked at week 7 after commencing vaccination regime but remained undetectable in unvaccinated group. |

| **Study** | **Study type** | **N** | **Intervention** | **Control group** | **HBV antigens within vaccine** | **Age group** | **Population** | **FU** | **Virologic assessment** | **Immunologic assessment** |
| --- | --- | --- | --- | --- | --- | --- | --- | --- | --- | --- |
| (Ishikawa and Kakumu, 2007) | Randomised | 53 | HBsAg with NUC | NUC | Pre-S2, S | Adult | HBeAg positive or negative chronic hepatitis | 52 | **HBV DNA suppression:** no significant difference between groups  **HBeAg loss:** no significant difference between groups **HBeAg seroconversion:** no significant difference between groups **HBsAg loss:** not reported | Not reported |
| (Hoa et al., 2009) | Randomised | 180 | Sci-B-Vac | NUC | PreS1, Pre-S2, S | Adult | HBeAg positive chronic hepatitis | 78 | **HBV DNA suppression:** Significantly reduced in groups treated with NUC **HBeAg loss:** no significant difference between groups **HBeAg seroconversion:** Significantly increased in groups treated with NUC **HBsAg loss:** no significant difference between groups | **HBs antibody levels:** detectable in n=50/120 (42%) in combine vaccination groups and n=0/60 (0%) in NUC monotherapy group. |
| (Lee et al., 2015) | Non-randomised | 32 | Hepavax‐Gene TF | No treatment | S | Adult | HBeAg negative chronic infection | 52 | **HBV DNA suppression:** no significant difference between groups **HBeAg loss:** not reported **HBeAg seroconversion:** not reported **HBsAg loss:** no significant difference between groups | Not reported |
| (Lai et al., 2018) | Non-randomised | 20 | ENGERIX-B | No control group | Pre-S2, S | Adult and child | HBeAg negative chronic infection | 96 | **HBV DNA suppression:** not reported **HBeAg loss:** not reported **HBeAg seroconversion:** not reported **HBsAg loss:** occurred in n=4/19 (21%) of vaccinated persons | Not reported |
| (Al-Mahtab et al., 2013) | Non-randomised | 20 | HBsAg + HBcAg | No treatment | S, C | Adult | Chronic | 48 | **HBV DNA suppression:** undetectable HBV DNA occurred in n=9/20 (45%) of vaccination group **HBeAg loss:** occurred in n=7/11 (64%) of vaccination group **HBeAg seroconversion:** occurred in n=2/11 (18%) of vaccination group **HBsAg loss:** not reported | **Cytokine ELISA (culture supernatant after in-vitro expansion)**: Increased cytokine production to HBV antigen stimulation in vaccinated compared to unvaccinated groups. |
| (Al Mahtab et al., 2018) | Randomised | 160 | NASVAC (HBsAg +HBcAg) | IFN | S, C | Adult | HBeAg positive or negative chronic hepatitis | 72 | **HBV DNA suppression:** significantly lower mean DNA in vaccinated group compared to control group at 24 week follow up after treatment completion **HBeAg loss:** not reported **HBeAg seroconversion:** occurred in n=5/14 (36%) of vaccinated group and n=3/16 (19%) of control group **HBsAg loss:** not reported | Not reported |

**Table S2 Immune complex vaccines**

| **Study** | **Study type** | **N** | **Intervention** | **Control group** | **HBV antigens within vaccine** | **Age group** | **Population** | **FU** | **Virologic assessment** | **Immunologic assessment** |
| --- | --- | --- | --- | --- | --- | --- | --- | --- | --- | --- |
| (Wen et al., 1995) | Non-randomised | 14 | HBVac + HBIG | No control group | S | Adult | HBeAg positive chronic infection or chronic hepatitis | 26 | **HBV DNA suppression:** occurred in n=9/14 (64%) **HBeAg loss:** occurred in n=6/14 (43%) **HBeAg seroconversion:** occurred in n=2/14 (14%) **HBsAg loss:** nil | Not reported |
| (Yao et al., 2007) | Randomised | 26 | YIC (HBsAg+ HBIG + allum adjuvant) | Placebo or no treatment | S | Adult | HBeAg positive chronic hepatitis | 44 | **HBV DNA suppression:** not reported **HBeAg loss:** in associated with 2 log reduction in HBV DNA titre occurred in n=5/10 (50%) vaccination group and n=0/10 (0%) control group **HBeAg seroconversion:** occurred in n=2/10 (20%) vaccinated and n=0/10 (0%) controls **HBsAg loss:** not reported | **HBs antibody levels:** detectable in n=2/10 (20%) vaccination group and n=0/10 (0%) control group **ICS:** detectable cytokine responses to HBsAg in n=4/5 (80%) of vaccine responders (HBeAg loss and HBV DNA reduction) and n=2/5 (40%) of vaccine non-responders |
| (Xu et al., 2008) and (Wang et al., 2010) | Randomised | 237/156 | YIC (HBsAg-HBIG + allum adjuvant) | Placebo | S | Adult | HBeAg positive chronic hepatitis | 44 | **HBV DNA suppression:** significantly lower in vaccine group if HBeAg seroconverted compared to non-seroconverted or control group. **HBeAg loss:** no significant difference b. **HBeAg seroconversion:** significantly higher in vaccinated compared to unvaccinated group **HBsAg loss:** significantly greater HBsAg decline in vaccine group if HBeAg seroconverted compared to non-seroconverted or control group. | Not reported |
| (Xu et al., 2013) | Randomised | 450 | YIC (HBsAg-HBIG + allum adjuvant) | Placebo | S | Adult | HBeAg positive chronic hepatitis | 76 | **HBV DNA suppression:** no significant difference between groups **HBeAg loss:** not reported **HBeAg seroconversion:** no significant difference between groups **HBsAg loss:** not reported | Not reported |

**Table S3 Lipopeptide epitope-based vaccines**

| **Study** | **Study type** | **N** | **Intervention** | **Control group** | **HBV antigens within vaccine** | **Age group** | **Population** | **FU** | **Virologic assessment** | **Immunologic assessment** |
| --- | --- | --- | --- | --- | --- | --- | --- | --- | --- | --- |
| (Heathcote et al., 1999) | Non-randomised | 90 | CY-1899 | No control group | Core (18-25)* | Adult | HLA-A*02 positive HBeAg positive or negative chronic infection or chronic hepatitis | 52 | **HBV DNA suppression:** no significant difference between baseline and after vaccination **HBeAg loss:** not reported **HBeAg seroconversion:** not reported **HBsAg loss:** not reported | **Cytotoxicity assay:** specific lysis increased in response to vaccination in a dose responsive way, however lower responses than in healthy vaccinated subjects |

**Table S4 DNA vaccines**

| **Study** | **Study type** | **N** | **Intervention** | **Control group** | **HBV antigens within vaccine** | **Age group** | **Population** | **FU** | **Virologic assessment** | **Immunologic assessment** |
| --- | --- | --- | --- | --- | --- | --- | --- | --- | --- | --- |
| (Mancini-Bourgine et al., 2004) and (Mancini-Bourgine et al., 2006) | Non-randomised | 10 | pCMV.PS2.S | No control group | Pre-S2, S | Adult | HBeAg positive chronic infection or chronic hepatitis | 44 | **HBV DNA suppression:** occurred in n=2/10 (20%) of vaccinated persons **HBeAg loss:** not reported **HBeAg seroconversion:** not reported **HBsAg loss:** not reported | **ELISpot (*ex-vivo)*:** baseline HBs responses in n=1/10 (10%). **ELISpot (after 14 days *in-vitro* expansion):** baseline HBs responses in n=2/10 (20%), increased to n=5/8 (63%) month 5 after vaccine **Proliferation assay:** No detectable responses at baseline. Detectable responses in n=2/10 (20%) after vaccination. |
| (Fontaine et al., 2015) | Randomised | 70 | pCMV-S2.S with NUC | NUC | Pre-S2, S | Adult | HBeAg positive or negative chronic infection | 72 | **HBV DNA suppression:** no significant difference between groups **HBeAg loss:** not reported **HBeAg seroconversion**: occurred in n=0/3 (0%) in vaccinated group and n=1/5 (20%) in control group **HBsAg loss:** occurred in n=2/34 (6%) in vaccinated group and n=1/36 (28%) in control group | **ELISpot (ex-vivo)**: Baseline responses present in 6-24% of participants. Increase in responses towards surface region after vaccination, but not significant difference between vaccinated group and control group. |
| (Cavenaugh et al., 2011) | Randomised | 64 | DNA pSG2.HBs and MVA vaccine (MVA.HBs). Vaccination alone or with NUC. | NUC or placebo (rabies vaccine) | Pre-S2, S | Adult | HBeAg positive or negative chronic infection | 48 | **HBV DNA suppression:** no significant difference between groups **HBeAg loss:** no significant difference between groups **HBeAg seroconversion:** nil **HBsAg loss:** nil | **ELISpot (*ex-vivo*):** low numbers of baseline interferon gamma producing cells, did not increase after vaccination |

| **Study** | **Study type** | **N** | **Intervention** | **Control group** | **HBV antigens within vaccine** | **Age group** | **Population** | **FU** | **Virologic assessment** | **Immunologic assessment** |
| --- | --- | --- | --- | --- | --- | --- | --- | --- | --- | --- |
| (Yang et al., 2012) | Randomised | 39 | ED-DNA.PS2.S with NUC | NUC + placebo | Pre-S2, S | Adult | HBeAg positive chronic hepatitis | 72 | **HBV DNA suppression:** decrease of >2 log from baseline achieved in significantly more in vaccinated group compared to control group **HBeAg loss:** no significant difference between groups **HBeAg seroconversion:** no significant difference between groups **HBsAg loss:** nil | **ELISpot (ex-vivo):** Positive responses in vaccinated group compared to control group at week 52 and week 72 of study.  **Flow cytometry (ex-vivo, HBV specific tetramers):** interferon gamma secreting T‐cell responses in n=6/9 (22%) vaccine+ NUC compared to n=2/7 (29%) in placebo + NUC group. |
| (Yang et al., 2017) | Randomised | 225 | ED-DNA.PS2.S with NUC | NUC + placebo | Pre-S2, S | Adult | HBeAg positive chronic hepatitis | 72 | **HBV DNA suppression:** no significant difference between groups **HBeAg loss:** not reported  **HBeAg seroconversion:** in participants with HBV DNA <1000 copies/mL at start of treatment, n=6/11 (55%) compared to n=3/19 (16%) seroconverted HBe  **HBsAg loss:** nil | **ELISpot (after 9 day in-vitro expansion):** detectable responses significantly increased n=19/22 (86%) in vaccine group compared to n=13/23 (57%) in control group.  **ICS:** Detectable CD8+ T cell responses significantly increased in the vaccine group n=17/22 (77%) compared to n=10/23 (44%) in control group. |
| (Yang et al., 2006) | Non-randomised | 12 | HB-100 with NUC | No control group | Pre-S1/Pre-S2/X, S, C, P | Adult | HBeAg positive or negative chronic hepatitis | 104 | **HBV DNA suppression (termed viral responders):** occurred in n=6/12 (50%) at end of treatment (week 52) **HBeAg loss**: not reported  **HBeAg seroconversion:** in n=4/6 (67%) **HBsAg loss:** occurred in n=1/12 (8%) | **ELISpot (ex-vivo):** No detectable responses at baseline, by end of treatment (week 52) responses to at least one HBV antigen were present in n=7/7 (100%) subjects tested. Responses significantly higher in viral responders compared to non-responders. By 40 weeks after treatment responses again undetectable ex-vivo. **ELISpot (after in-vitro expansion):** Detectable responses 40 weeks after in viral responders. Most responses CD4+ (using PBMC depletion of CD4/8 prior to ELISpot assay). |
| (Yoon et al., 2015) | Randomised | 27 | HB-110 with NUC | NUC | Pre-S2, S, C, P | Adult | HBeAg negative chronic hepatitis | 48 | **HBV DNA suppression:** no significant difference between groups **HBeAg loss**: not reported **HBeAg seroconversion:** occurred in n= 0/7 (0%) in vaccinated and n=4/18 (22%) in controls **HBsAg loss:** not reported | **ELISpot (*ex-vivo*):** Detectable responses in n=5/18 (28%) of vaccinated group and n=0/6 (0%) control group. **Proliferation assay:** Vaccine induced T cells were predominantly CD8 and polyfunctional |

**Table S5 Yeast derived vaccines**

| **Study** | **Study type** | **N** | **Intervention** | **Control group** | **HBV antigens within vaccine** | **Age group** | **Population** | **FU** | **Virologic assessment** | **Immunologic assessment** |
| --- | --- | --- | --- | --- | --- | --- | --- | --- | --- | --- |
| (Lok et al., 2016) | Randomised | 178 | GS-4774 with NUC | NUC | S, C, X | Adult | HBeAg positive or negative chronic infection | 48 | **HBV DNA suppression:** no HBV DNA breakthrough in any group **HBeAg loss:** occurred in n=5/37 (14%) of vaccinated groups and n=0/7 (0%) of control group **HBeAg seroconversion:** occurred in n=4/37 (11%) of vaccinated groups and n=0/7 (0%) of control group **HBsAg loss:** no HBsAg loss in any group, no significant difference in quantitative HBsAg levels between vaccinated and unvaccinated groups | **ELISpot (*ex-vivo*):** positive response to HBcAg in n=53/94 (56%) in vaccinated groups and n=5/14 (36%) in control group |
| (Gane et al., 2019) | Non-randomised | 24 | GS-4774 with NUC | Nivolumab with NUC | S, C, X | Adult | HBeAg negative chronic infection | 24 | **HBV DNA suppression:** not reported **HBeAg loss:** not reported **HBeAg seroconversion:** not reported **HBsAg loss:** occurred in n=0/10 (0%) vaccinated group and n=1/14 (7%) control group | **ELISpot (*ex-vivo*):** detectable responses at baseline towards HBc and HBs, no change in response to vaccination. The individual that lost HBsAg during the study had the highest magnitude of T cell responses. |
| (Boni et al., 2019) | Randomised | 195 | GS-4774 with NUC | NUC | S, C, X | Adult | HBeAg positive or negative chronic hepatitis | 48 | **HBV DNA suppression:** not reported **HBeAg loss:** occurred in n= 5/66 (8%) in vaccinated groups and n= 0/10 (0%) in control group **HBeAg seroconversion:** occurred in n= 3/66 (8%) in vaccinated groups and n= 0/10 (0%) in control group **HBsAg loss:** nil HBsAg loss and no significant difference in quantitative HBsAg decline between groups | **ELISpot (ex-vivo):** Increased T cell responses compared to baseline in vaccinated group but not in control group **Proliferation assay and ICS:** Significantly increased cytokine production by HBV specific CD8 T cells in vaccinated compared to unvaccinated groups |

**Table S6 Adenoviral vectored vaccines**

| **Study** | **Study type** | **N** | **Intervention** | **Control group** | **HBV antigens within vaccine** | **Age group** | **Population** | **FU** | **Virologic assessment** | **Immunologic assessment** |
| --- | --- | --- | --- | --- | --- | --- | --- | --- | --- | --- |
| (Zoulim et al., 2020) | Randomised | 48 | Human adenoviral type 5 vector (TG1050) with NUC | Placebo with NUC | S, C, P | Adult | HBeAg positive or negative chronic infection | 54 | **HBV DNA suppression:** not reported **HBeAg loss:** not reported  **HBeAg seroconversion:** not reported **HBsAg loss:** nil HBsAg loss | **ELISpot (*after 10 days of in-vitro expansion*):** detectable responses increased after vaccination compared to baseline in n=9/17 (53%) of vaccinated group but no responses were detectable above baseline in the control group |
